# Supplementary material for: Chromothripsis during telomere crisis is independent of NHEJ, and consistent with a replicative origin
Source: Genome Res. 2019 May;29(5):737–49. doi: 10.1101/gr.240705.118 (PMC6499312; doi:10.1101/gr.240705.118)
Supplement: Supplemental Material [file supp_gr.240705.118_Supplemental_file_1.zip › contigs/annotated_contigs/DB49/contig.3.DB49_length_533_mean_cov_6.43902439024.docx]

**DB49_length_533_mean_cov_6.43902439024**

```
TTCAGACCACACAATTTCCAAAAAGCCAAAAACTTAT|CAAAAACTAGTTTTCTGGAGTTGGGAGAATGAGGAAGATTCAATATTCCAA
                                      >chrUn_gl000225:159384-159463 + E=8e-09
GTGACCTTGGTGGCATTTCATAGCTCAA|AAAAAAAAAAA|AAAAATTTTATTCCTTTTTCATTTCTGTTAGGGAATGTTTAGTTTTTC
                                        >chrY:59017441-59017798 + E=1e-181 p=0e+00
CTCTTTGATAATGAAAATTTTGTTAACAAACATTGCTTTGGACCAATCTTCCTTTTCACAAATCACTGAGGTGTGTCTATCCACAAGTT

CTCAGTTCAGCCTACATTTTCCAGTCTTCCATTTACCTATTCAACTAATACCTGAGTGCCTGGCATGTGTCAGCCCTGTGAACATGCTG

GTGACAGTGTGAGCAGGCAGGGCTGGTTCCTGTCCTCATGAAGCTTATACTACCTGAGGCTGGGGTGTAGAAATCGACACATTAAAAAG

TCTACAGAGACTTTAAAATATATATATATATATATAT|ATATATA|TATATATATATATATATATATATATATATATATATCTTTAACA
                                             >chr5:24303166-24303210 + E=8e-12
T|GAT
```
